# New insights in ubiquitin-dependent Wnt receptor regulation in tumorigenesis

**DOI:** 10.1007/s11626-024-00855-w

**Published:** 2024-02-21

**Authors:** Tadasuke Tsukiyama

**Affiliations:** Department of Biochemistry, Graduate School of Medicine, Hokkaido University, 15NW7, Kita-Ku, Sapporo, Hokkaido 060-8638 Japan

**Keywords:** Wnt, Fzd, Ubiquitin, RNF43, Tumorigenesis

## Abstract

Wnt signaling plays a crucial role in embryonic development and homeostasis maintenance. Delicate and sensitive fine-tuning of Wnt signaling based on the proper timings and positions is required to balance cell proliferation and differentiation and maintain individual health. Therefore, homeostasis is broken by tissue hypoplasia or tumor formation once Wnt signal dysregulation disturbs the balance of cell proliferation. The well-known regulatory mechanism of Wnt signaling is the molecular reaction associated with the cytoplasmic accumulation of effector β-catenin. In addition to β-catenin, most Wnt effector proteins are also regulated by ubiquitin-dependent modification, both qualitatively and quantitatively. This review will explain the regulation of the whole Wnt signal in four regulatory phases, as well as the different ubiquitin ligases and the function of deubiquitinating enzymes in each phase. Along with the recent results, the mechanism by which RNF43 negatively regulates the surface expression of Wnt receptors, which has recently been well understood, will be detailed. Many RNF43 mutations have been identified in pancreatic and gastrointestinal cancers and examined for their functional alteration in Wnt signaling. Several mutations facilitate or activate the Wnt signal, reversing the RNF43 tumor suppressor function into an oncogene. RNF43 may simultaneously play different roles in classical multistep tumorigenesis, as both wild-type and mutant RNF43 suppress the p53 pathway. We hope that the knowledge obtained from further research in RNF43 will be applied to cancer treatment in the future despite the fully unclear function of RNF43.

## Introduction

Wnts are secreted lipid-modified glycoproteins, and they constitute a large family with 19 members in humans (Buechling and Boutros 2011; Dijksterhuis *et al*. 2014; Van Camp *et al*. 2014). Wnt proteins transduce several signals within a very short distance from Wnt-secreting cells due to their strong hydrophobicity (Farin *et al*. 2016). Ten Wnt receptors, Frizzleds (Fzds), are expressed on the Wnt-receiving cells and coupled with co-receptors, such as LRP5/6 or Ror1/2, to transduce signals into cells (Kikuchi *et al*. 2009). When Wnt ligands form ligand-receptor complexes with Ror1/2, the Wnt-Fzd-Ror complexes transduce noncanonical signals via Wnt/PCP or Wnt/Ca^2+^ pathways and control cell polarity and motility (Kikuchi *et al*. 2012). Alternatively, ligand-receptor complexes with LRP5/6 activate the canonical Wnt signal and cause the expression of a series of Wnt target genes via the accumulation of a key effector, β-catenin. The canonical Wnt/β-catenin signal maintains cell stemness and proliferation in an undifferentiated state (MacDonald and He 2012). The combination of Wnt ligands-receptor-co-receptor intricately regulates cellular behaviors, such as how cells proliferate and move.

Wnts are assumed to function in both embryonic development and tumorigenesis based on the history of Wnt signal discovery. In the 1970s, a fly mutant with no wings due to the segment polarity defect was identified (Sharma and Chopra 1976; Nusslein-Volhard and Wieschaus 1980). This was named wingless mutant. Independently of these reports in the developmental biology field in 1982, another group studying virus-induced tumorigenesis revealed that the virus is frequently inserted in the genomic region, Int-1, when mouse mammary gland tumor virus was used to infect to mice for tumor formation (Nusse and Varmus 1982). In 1987, these were identified with the same gene, and *Int-1* was reported as the mouse homolog of fly *wingless* gene (Baker 1987; Rijsewijk *et al*. 1987). Therefore, this locus was named *Wnt-1* by taking letters from *wingless* and *int-1*, as well as wingless-related integration sites.

The study that explored the biological function of Wnt started with fly, but now, the roles of most Wnts have been dissected in detail using gene disrupted mice model. Individual phenotypes of Wnt-deficient mice are listed on “the Wnt homepage” (https://web.stanford.edu/group/nusselab/cgi-bin/wnt/). Several studies revealed that Wnts play a crucial role in determining body plan during early embryogenesis (Yamaguchi 2008; Schnirman *et al*. 2023).

Wnts play important roles even in adults who have completed their development and growth. It is generally thought that Wnt/β-catenin signaling activity is indispensable for stem cell maintenance, particularly in maintaining tissue homeostasis in the gastrointestinal (GI) tract, hematopoietic system, and skin, where cell turnover is high.

The perturbation of Wnt signaling is closely associated with human diseases (Logan and Nusse 2004; Clevers and Nusse 2012). Genetic mutations in Wnt ligands, Fzds, and co-receptors frequently cause congenital hypoplasia in hard tissues, such as the bones and tooth, or bone maintenance in adults (see the Wnt homepage). Furthermore, canonical Wnt/β-catenin signal dysregulation strongly induces tumorigenesis especially in the GI tract (Zhan *et al*. 2017; Koushyar *et al*. 2020; Zhu and Li 2023). Genetic analyses with mutant flies, a tumorigenic adenomatous polyposis coli-min (APC^min^) mutant mice, and patients with familial adenomatous polyposis (FAP) allowed us to understand how the failure of β-catenin degradation by loss of function (LOF) of *APC* triggers colorectal cancer (CRC).

This review will explain the Wnt receptor degradation mechanism, which has been rapidly understood in the last 10 yr, and explore the progression of carcinogenesis due to its failure, using the latest results.

## Wnt/β-catenin signaling regulation is categorized into four phases

### First phase: From the Wnt protein synthesis to its secretion outside of Wnt-producing cells via intracellular trafficking

**Figure 1.**
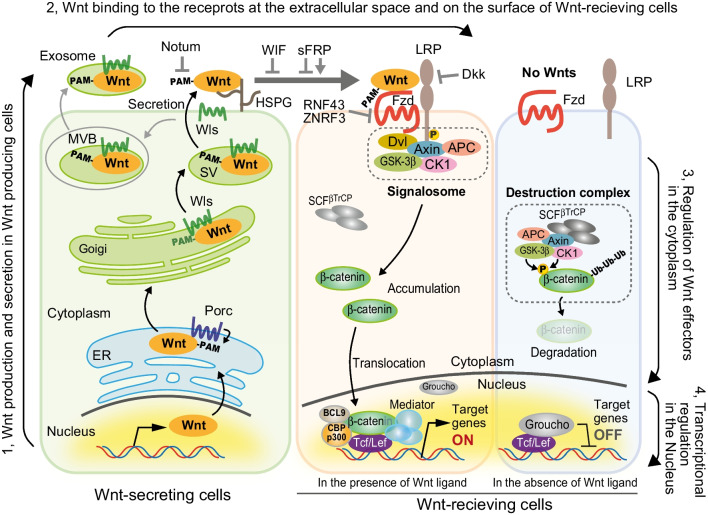
Four regulatory phases of Wnt signal transduction. Whole Wnt signal cascades consist of four identical regulatory events: (1) how much Wnt-producing cells secret Wnt ligand proteins to outside of the cells (*left side*); (2) how much Wnt-receiving cells are stimulated by functional Wnt ligands at the extracellular and receptors on the cell surface (*top*); (3) how Wnt-received cells intracellularly transduce the signals to the downstream points of action (*right side*, *upper*); (4) how cells respond to the signal received via several biological reactions, including the expression of Wnt target genes (*right side*, *bottom*). ER, endoplasmic reticulum; PAM, palmitoleic acid; MVB, multivesicular body; SV, secretory vesicle; Ub, ubiquitin.

This phase is essentially regulated by the amount of secreted Wnt ligand proteins. Wnt proteins are synthesized in the endoplasmic reticulum (ER), modified with lipids, and then secreted outside cells via trans-Golgi network (TGN) and secretory vesicles (SVs) or multivesicular bodies (MVBs) (Fig. 1, *left*) (Hosseini *et al*. 2019; Routledge and Scholpp 2019). During this process, Wnt proteins are modified with palmitoleic acid (PAM) in a conserved serine by ER membrane–expressing protein-serine O-palmitoyltransferase, porcupine (PORCN) (Kadowaki *et al*. 1996; Takada *et al.* 2006). The PAM modification makes Wnt proteins hydrophobic and directs the binding to a cavity of Wntless (Wls/Evi) located in transmembrane regions (Nygaard *et al*. 2021; Zhong *et al*. 2021). Wls-bound Wnts are transported via the intracellular membrane trafficking pathway for their extracellular secretion (Banziger *et al*. 2006). Therefore, PAM modification of Wnt is a crucial essential process in Wnt signaling regulation. Wnts that lack PAM cannot bind to Wls; thus, they are not secreted extracellularly. Similar to gene mutations in *PORCN*, PORCN activity inhibition by small molecules (PORCNi) or *Wls* mutation also lacks Wnt secretion and the Wnt signaling activation (van den Heuvel *et al*. 1993; Barrott *et al*. 2011; Proffitt *et al*. 2013).

### Second phase: Until secreted extracellular Wnts bind to their receptors on the Wnt-receiving cell

This phase involves multiple regulation mechanisms, including Wnt activity, extracellular travels, and binding and level of receptors and co-receptors. Secreted Wnt proteins are released from Wls and are highly hydrophobic. PAM-modified Wnts cannot travel even in short distances from Wnt-producing cells if they remain naked. Therefore, the stable migration of Wnts is believed to occur in various mechanisms (Fig. 1, *top*) (Takada *et al.* 2017; Mehta *et al*. 2021). The secretion of exosomes containing Wnts via MVBs can protect Wnt proteins and keep them stable in extracellular space (Gross *et al*. 2012). The adhesion of Wnt protein to heparan sulfate proteoglycans (HSPGs) that are expressed on the cell surface enables Wnts to exist stably and migrate across the cell surface (Binari *et al*. 1997; Yan and Lin 2009). Extracellular proteins, such as Swim, lipoprotein particle (LPP), and secreted frizzled-related proteins (sFRPs), bind to Wnts to carry them (Panakova *et al*. 2005; Neumann *et al*. 2009; Mulligan *et al*. 2012). According to their Wnt binding ability, sFRPs were initially accepted as Wnt agonists (Finch *et al*. 1997). Additionally, sFRPs were thought to interact with Wnts via cysteine-rich domain (CRD) that is also conserved in Wnt receptor Fzds and competes with Wnt-Fzd binding (Kawano and Kypta 2003). However, sFRPs have been recently found to have two sides in Wnt signal regulation. sFRPs directly bind to Wnts, help them to be soluble and stable in extracellular space, and carry Wnt proteins to distant Wnt-receiving cells by expanding the range of Wnt action (Mii and Taira 2009; Esteve *et al*. 2011). Conversely, several “pure” inhibitor proteins of the Wnt signal are also acting in extracellular space. The addition of PAM modification was explained to be essential for Wls binding to and extracellular secretion of Wnt proteins. Further, PAM is essential for Wnt binding to Fzd to activate intracellular downstream signaling (Hirai *et al*. 2019; Janda *et al*. 2012). However, Notum impairs the activity of Wnt proteins by deacylating and removing PAM from extracellularly secreted Wnts (Kakugawa *et al*. 2015). In addition to these Wnt-affecting proteins, some proteins also act inhibitory on the Wnt receptors and co-receptors. Dickkopf-1 (Dkk-1) interacts with co-receptors and low-density lipoprotein receptor-related proteins 5 and 6 (LRP5/6) and blocks Wnt-LRPs binding to avoid Wnt/β-catenin signaling activation (Glinka *et al*. 1998; Mao *et al*. 2001; MacDonald and He 2012). Membrane-bound ubiquitin ligases, RNF43 and ZNRF3, degrade Wnt receptor Fzds in a ubiquitination-dependent manner, thereby downregulating the surface expression of receptors and reducing the sensitivity to Wnt ligands (Hao *et al*. 2012; Koo *et al*. 2012; de Lau *et al*. 2014; Farnhammer *et al*. 2023).

### Third phase: Wnt effector molecule activation in the cytoplasmic region

This phase is the best-known regulation in the Wnt signaling pathway and essentially regulates by the amount of β-catenin protein. The intracellular destruction complex, consisting of APC, Axin, GSK-3β, and CK1, constitutively phosphorylates β-catenin in the absence of Wnt ligands (Fig. 1, *right*) (Logan and Nusse 2004; Clevers and Nusse 2012; Nusse and Clevers 2017). Phospho-β-catenin is recognized and ubiquitinated by a ubiquitin ligase complex SCF^β−TrCP^ (including Cul-1, Skp1, and β-TrCP) and rapidly downregulated by proteasomal degradation, resulting in the limited free β-catenin in the cytoplasm (Jiang and Struhl 1998; Kitagawa *et al*. 1999; Latres *et al*. 1999). However, the intracellular serines of LRPs are phosphorylated by GSK-3β and/or CK1 once Wnt ligands bind to Fzds and LRPs, and they then interact with Axin (Fig. 1, *middle*). Fzd and Axin start to polymerize via the DIX domain of Dishevelled (Dvl) and DAX domain of Axin and form a large signalosome that inhibits GSK-3β activity just under Fzd and LRP (Roberts *et al*. 2007; Fiedler *et al*. 2011; Kan *et al*. 2020; Beitia *et al*. 2021). GSK-3β inactivation disables to phosphorylate β-catenin and allows it to escape from SCF^β−TrCP^-mediated degradation, resulting in the raid and drastic β-catenin accumulation. These excess cytoplasmic unphospho-β-catenin proteins start to translocate into the nucleus.

### Forth phase: Transcriptional activation of Wnt target genes in the nucleus

This phase is essentially regulated by the interaction of Tcf/Lef with Groucho or β-catenin on the Wnt-responsive elements (WREs). A transcriptional suppressor Groucho keeps the interaction with a family of HMG-box transcription factor Tcf/Lef proteins located on WREs when the Wnt ligands are absent and β-catenin proteins are not accumulated in the cytoplasm and the nucleus (Fig. 1, *lower right*) (Cavallo *et al*. 1998; Daniels and Weis 2005; Cadigan and Waterman 2012). Therefore, Groucho strongly suppresses Wnt target genes whose expression is regulated via Tcf/Lef transcription factors, when deubiquitinated by USP47 (Kassel *et al*. 2023). However, Groucho dissociates with Tcf/Lef on WRE by X-linked inhibitor of apoptosis (XIAP)–dependent ubiquitination once Wnt ligands activate Wnt/β-catenin signaling (Hanson *et al*. 2012). Abundant β-catenin binds to Tcf/Lef and replaces Groucho (Fig. 1, *middle-bottom*). The binding of β-catenin to Tcf/Lef on the WRE recruits several factors that initiate and activate the Wnt target gene transcription, including CBP/p300 for chromatin remodeling, BCL9 to cooperate with enhancers, and mediator complex to initiate the transcription (Anthony *et al*. 2020). A classical oncogene c-myc, which can induce cell stemness, plays a central role in tumorigenesis among the many Wnt target genes (Shachaf *et al*. 2004; Takahashi and Yamanaka 2006; Sansom *et al*. 2007).

Here, we describe the essential roles of ubiquitin-dependent modification in regulating various Wnt effector proteins’ qualitative and quantitative activity, especially in Wnt-receiving cells.

## Ubiquitination rules Wnt signaling

**Figure 2.**
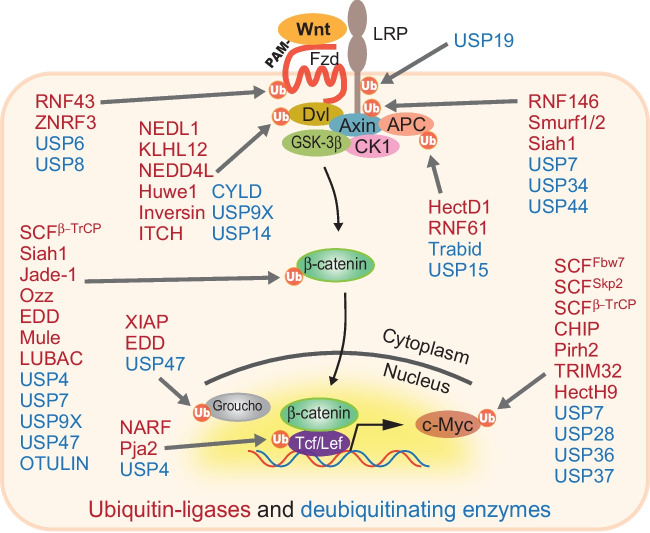
Ubiquitin-dependent proteolysis governs Wnt signaling. A large number of ubiquitin ligases (shown in *red*) and deubiquitinating enzymes (shown in *blue*) quantitatively and qualitatively regulate most of the effectors that are involved in Wnt signaling. Multiple ligases and deubiquitinating enzymes are assigned to each effector. Some other enzymes for ubiquitin-dependent modification may also have been identified, but not all are shown here.

The majority of Wnt effector proteins, particularly in Wnt-receiving cells, are functionally and quantitatively regulated by ubiquitination-dependent modification to fine-tune the signal to keep it in the state of dynamic equilibrium. These effector ubiquitinations are sometimes cooperated with other modifications, including phosphorylation (Gao *et al*. 2014). Furthermore, each effector does not have a one-to-one correspondence with its ubiquitinating or deubiquitinating enzymes but is regulated by multiple groups of these modifying enzymes in a complex and context-specific manner (Fig. 2) (Tauriello and Maurice 2010; Deng *et al*. 2020).

Wnt receptors and co-receptors function as an entrance of the signal at the most upstream of this cascade. Co-receptor LRP6 is deubiquitinated by USP19 to regulate its protein maturation and expression on the cell surface, whereas its ubiquitin ligases remained unidentified (Perrody *et al*. 2016). The Wnt receptor Fzd surface level is regulated by two membrane-bound ubiquitin ligases, RNF43 and ZNRF3. ZNRF3 deubiquitination by USP42 stabilizes its surface retention and functions to downregulate Fzds (Hao *et al*. 2012; Koo *et al*. 2012; Colozza and Koo 2021; Giebel *et al*. 2021). Conversely, Fzd is deubiquitinated by USP6 and USP8 (Mukai *et al*. 2010; Jung *et al*. 2013; Madan *et al*. 2016). Dvl is ubiquitinated by NEDL1, KLHL12, NEDD4L, Huwe1, Inversin, and ITCH and deubiquitinated by CYLD, USP9X, and USP14 (Miyazaki *et al*. 2004; Angers *et al*. 2006; Tauriello *et al*. 2010; Wei *et al*. 2012; Ding *et al*. 2013; Jung *et al*. 2013; de Groot *et al*. 2014; Nielsen *et al*. 2019). A component of the destruction complex, Axin, is ubiquitinated by RNF146, Smurf1/2, and Siah1 and deubiquitinated by USP7, USP34, and USP44 (Kim and Jho 2010; Lui *et al*. 2011; Zhang *et al.* 2011; Fei *et al*. 2013;  Ji *et al.* 2017; Ji *et al.* 2019; Huang *et al.* 2020). APC is ubiquitinated by HectD1 and RNF61 and deubiquitinated by Trabid and USP15 (Tran et al. 2008; Huang *et al.* 2009; Tran *et al*. 2013; Lee *et al*. 2018). A core effector, β-catenin, is also modified by multiple enzymes, including ligases SCF^β−TrCP^, Siah1, Jade-1, Ozz, EDD, Mule, and LUBAC, and deubiquitinated by USP4, USP7, USP9X, USP47, and OTULIN (Kitagawa *et al*. 1999; Liu *et al*. 2001; Nastasi *et al*. 2004; Chitalia *et al*. 2008; Hay-Koren *et al*. 2011; Shi *et al*. 2015; Yun *et al*. 2015; Ouyang *et al*. 2016; Dominguez-Brauer *et al*. 2017; Novellasdemunt *et al*. 2017; Wang *et al*. 2020; Zhang *et al.* 2021).

Tcf/Lef transcription factors, which function as scaffolds to recruit β-catenin to WRE on target genes, are regulated by NARF, Pja2, and USP4 in the nucleus (Yamada *et al*. 2006; Zhao *et al*. 2009; Song *et al*. 2018). Additionally, the Groucho corepressor, which inhibits Wnt target activation while the Wnt signal is off, is regulated by XIAP, EDD, and USP47 (Hanson *et al*. 2012; Flack *et al*. 2017; Kassel *et al*. 2023). Finally, a critical Wnt target in tumorigenesis, c-myc, is regulated by a more complicated mechanism as reviewed in detail (Sun *et al.* 2021). C-myc is ubiquitinated by at least 18 ligases, including SCF^β−TrCP^, SCF^Fbw7^, SCF^Skp2^, CHIP, Pirh2, TRIM32, and HectH9, and 6 deubiquitinases, including USP7, USP28, USP36, and USP37 (von der Lehr *et al*. 2003; Yada *et al*. 2004; Adhikary *et al*. 2005; Popov *et al*. 2007; Popov *et al*. 2010; Hakem *et al*. 2011; Paul *et al*. 2013; Pan *et al*. 2015; Sun *et al.* 2015; Nicklas *et al*. 2019).

Generally, modification with K48-linked polyubiquitin chains caused proteasomal degradation, but not in other chain types. Ubiquitin-dependent modification of the abovementioned Wnt effectors not only induces Wnt effector protein degradation, but also stabilizes them by blocking the proteasomal degradation via other ubiquitin chain types on the effectors or regulating protein–protein interaction with other effectors or factors. Additionally, these ubiquitin-dependent modifications are sometimes coupled with other modifications including phosphorylation making signal regulation complex but highly elastic.

## Ubiquitination-dependent Wnt receptor regulation in co-operation with a growth factor

As explained above, the surface expression of Wnt receptors is also regulated by ubiquitin-dependent modification. The deubiquitinating enzyme of Fzd has been known for some time, but the ubiquitinating enzyme remained unknown for a time. However, several independent groups reported from 2012 to 2015 that two ubiquitin ligases, RNF43 and ZNRF3, which are specifically expressed in stem cells, are enzymes that lead Fzd degradation (Hao *et al*. 2012; Koo *et al*. 2012; Tsukiyama *et al*. 2015). A Japanese research group in 2004 first reported RNF43 as an oncoprotein that is highly expressed in CRC, but its function remained unclear for a long time (Yagyu *et al*. 2004). RNF43 is a type I single transmembrane protein with a transmembrane (TM) region and two functional domains, namely a protease-associated (PA) domain in the extracellular region for interacting with other proteins and an intracellular ring-finger (RING) domain for modifying proteins with ubiquitin. Wnt receptor regulation by RNF43/ZNRF3 is carried by a complex and beautiful mechanism that cooperates with an intestinal growth factor R-spondin (Rspo) (Hao *et al*. 2012; de Lau *et al*. 2014).

**Figure 3.**
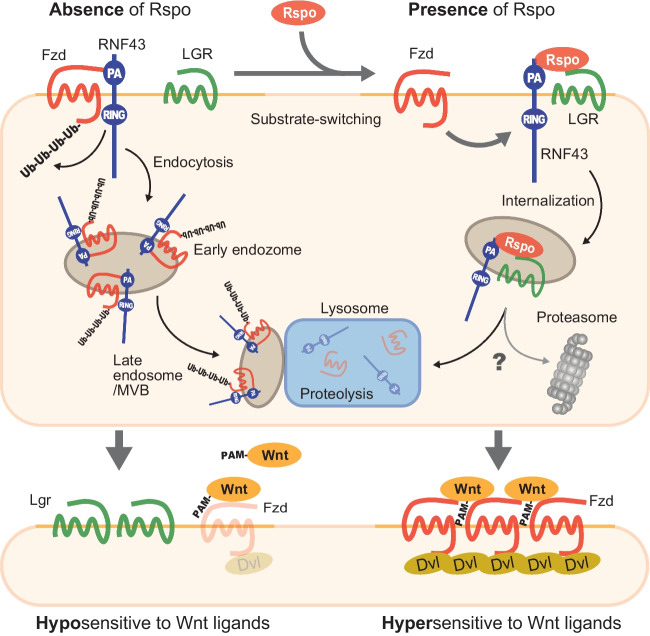
Mechanism by which Rspo accumulates Wnt receptors on the cell surface. RNF43 interacts with Wnt receptor Fzd in the absence of Rspo, to ubiquitinate and induce receptor internalization and lysosomal degradation (*upper left*), retaining Wnt-receiving cells in the hyposensitive state to Wnt ligands (*lower left*). Rspo deprives Fzds of RNF43 once cells are exposed to Rspo, and it forms RNF43-Rspo-LGR complex (*upper right*) to switch its substrates. Fzds escape from RNF43-derived degradation and start to accumulate on the cell surface, thereby acquiring the hyper-sensitivity to the same amount of Wnt ligands (*lower left* vs. *right*).

RNF43 interacts with Fzds, in the absence of Rspo, to ubiquitinate them. Proteasomal degradation even with ubiquitin modification does not downregulate Fzds, but ubiquitination initiates the internalization of Fzds and co-receptor LRP5/6 and lysosomal degradation via endosome (Fig. 3, *upper left*). Therefore, the Fzd expression level on the cell surface is kept quite low, and the cellular sensitivity to Wnt ligands is also maintained in low (Fig. 3, *lower left*). However, once Rspo reaches the area, it forms a trimeric complex with RNF43/ZNRF3 and LGR4/5 which is well-known as a stem cell marker by bridging these two proteins (Fig. 3, *upper right*). Rspo switches RNF43 substrates, and LGR4/5/6 internalization occurs instead of Fzd. As a result, Fzd escapes the degradation by RNF43/ZNRF3 and accumulates on the cell surface. Elevated Fzd expression on the cell surface drastically enhances the sensitivity to equal amounts of Wnt ligands in the presence of Rspo (Fig. 3, *lower right*). Amplification of signal by the change of surface receptor level can fine-tune the signal with a high signal/noise ratio in addition to a regulatory layer on how much Wnt ligands stimulate Wnt-receiving cells.

Furthermore, recent reports proposed distinct models between LGR4 and LGR5 in facilitating Wnt signaling that LGR4 forms complex with bivalent Rspo and RNF43/ZNRF3 as in the present model, whereas LGR5 and Rspo directly stimulate signalosome regardless of its valency regardless of these ubiquitin ligases via IQGAP1 (Park *et al*. 2020; Toh *et al*. 2023).

## The phospho-switch of RNF43, a novel regulatory mechanism in Fzd expression and Wnt signaling

**Figure 4.**
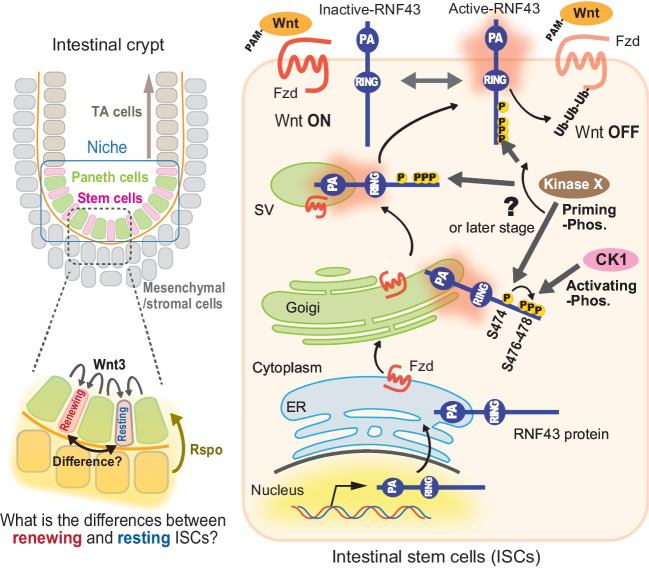
Phosphorylation-dependent functional RNF43 activation. Intestinal niches (*left*, *blue boxed*) are located at the bottom of intestinal crypts surrounded by mesenchymal and stromal cells. The niche consists of intestinal stem cells (ISCs) and Paneth cells. Paneth cells are derived from ISCs and secrete Wnt ligands toward adjacent ISCs to maintain stemness, whereas Rspo is secreted and diffuses from mesenchymal and/or stromal cells at the outside of intestinal niche (*left*, *dashed gray boxed*, and *bottom*). Post-translational RNF43 is activated by phosphorylations at some point (unidentified) during its trafficking from the Golgi to the cell surface and acquires the ability as a ubiquitin ligase (*right*). This activation occurs in at least two stages: unidentified kinase X firstly primes S474, and then, the following phosphorylation of three serines at 476**–**478 by CK1 activates RNF43.

The intestinal tract can be histologically categorized into villi and crypts. Intestinal epithelial cells that are generated and matured in the crypt move to the villi, and they fall off once they function well and reach the end of their lifespan, thereby maintaining intestinal homeostasis (Gehart and Clevers 2019). Intestinal stem cells (ISCs) live in the bottom of intestinal crypts with Paneth cells and form intestinal niches (Sato *et al*. 2011) (Fig. 4, *left upper*). Most ISCs do not proliferate in a quiescent state but a fraction of them proliferates slowly to maintain stem cells by self-renewal or to produce transient-amplifying (TA) cells. TA cells gradually differentiate while continuing proliferation, and various cells, including differentiated and growth-arrested epithelial cells, are pushed out of the crypt to the villi. Wnt signaling plays an essential role in ISC self-renewal and TA cell proliferation (Merenda *et al*. 2020). RNF43 is expressed only in the ISCs (Fig. 4, *left*, *lower*) (Koo *et al*. 2012). However, distinguishing between quiescent or proliferative stem cells in the same intestinal niche may be difficult because Wnt proteins are secreted from adjacent Paneth cells and act only at very short distances. Unlike Wnts, Rspo is secreted from stromal cells that surround the intestinal niche, so they are thought to be more diffusible and widespread than Wnts (Farin *et al*. 2016). Therefore, the additional regulatory layer is speculated to be required to fine-tune the signal in stem cells in the niche.

The regulation of function by RNF43 phosphorylation answers this speculation. RNF43 is believed to be transported to the cell surface by intracellular membrane trafficking pathway via ER, TGN, and MVBs/SVs after protein synthesis (Fig. 4, *right*). Nascent RNF43 proteins are not phospho-modified, but a serine cluster consisting of conserved four serines in the cytoplasmic region is phosphorylated at some time/some place after Golgi. These serines are phosphorylated localization-dependently in multi-steps. Priming phosphorylation on S474 (by unidentified kinase X) causes sequential phosphorylation on the S476/477/478 cluster by casein kinase I (CK1) to activate the function of RNF43 as a ubiquitin ligase and turning the “switch to ON” (Tsukiyama *et al*. 2020). After RNF43 activation by these serine phosphorylations, CK1 further phosphorylates RNF43, although the role of hyper-phosphorylation remains unknown.

The phosphorylation of RNF43 bidirectionally regulates Wnt signaling via Fzd expression on the cell surface, so it was defined as the “phospho-switch.” RNF43 fully retained the function when the phospho-switch that is fixed in the ON by core serines was substituted with aspartic acids or glutamic acids (3SD or 3SE) for mimicking serine phosphorylation. RNF43 (3SD/3SE) can ubiquitinate and downregulate Fzds to suppress Wnt signaling. Conversely, replacing these serines with arginines (3SA) to mimic unphosphorylated serines and fixing the switch to OFF cause the RNF43 to lose its function. RNF43 (3SA) facilitates Wnt signaling due to surface Fzd accumulation. Therefore, the phospho-switch is thought to maintain homeostasis by balancing differentiation and proliferation by fine-tuning Wnt signaling. Indeed, normal intestinal tissues cannot be maintained due to reduced Wnt signaling caused by excessive membrane clearance of Fzds when fixing in the switch to ON with 3SD (cannot be turned off). Meanwhile, tumors are induced by an excess of Fzd and Wnt signaling in the phospho-switch constitutively OFF state with 3SA (cannot be turned on). The phospho-switch finding answers how stem cells in the same niche that are exposed to both Wnt ligands and Rspo similarly can make the difference between whether to proliferate or arrest (Fig. 4, *left lower*). Phosphorylation activates the function of RNF43, but the dephosphorylating enzyme that inactivates and turns the switch off remains unidentified. Further, the kinase responsible for initially and actively triggering the switch remains unknown despite identifying CK1 as a kinase activating RNF43 in the second step of the phospho-switch. Therefore, identifying phosphatases and kinases and their upstream signals is a significant step toward understanding bidirectional Wnt signal regulation by the phospho-switch. Furthermore, the phosphorylation status of four tyrosines on ZNRF3 (not conserved in RNF43) by protein tyrosine phosphatase receptor-type kappa and mesenchymal-epithelial transition factor reported recently to determine its localization to the cell surface, thereby regulating Wnt signals via Fzd expression (Nanki *et al*. 2018; Chang *et al*. 2020). This regulatory mechanism also functions as another “phospho-switch” in Fzd and Wnt signal regulation. Several mysteries remain regarding the relationship between the phosphorylation mechanism of RNF43/ZNRF3 and its subcellular localization. Further research is needed to understand fully the function of RNF43/ZNRF3.

## Mutations of *RNF43* in tumorigenesis

**Figure 5.**
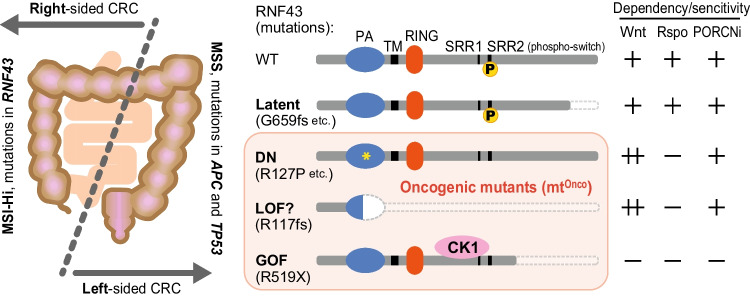
Classification of RNF43 mutations in cancers. The colon can be divided into two major parts, the left side or the right side, based on the difference in their developmental origin (*left*). The nature of the cancer and the combination of their genetic mutations also differ greatly between these parts. Mutations that are found in cancers are firstly classified into two (*right*). One is the “latent mutations” without impact in Wnt signal suppression and functions similar to wild-type. The other is the “oncogenic mutations” (*pink boxed*). This class is categorized into three subgroups, DN, LOF, or GOF mutations. However, all mutations belonging to these subgroups facilitate Wnt signaling and convert RNF43 tumor suppressor into oncogene in distinct mechanisms. CRC, colorectal cancer; MSS, microsatellite stable; MSI-Hi, microsatellite instability-high; DN, dominant-negative; LOF, loss of function; GOF, gain of function; PA, protease-associated domain; TM, transmembrane region; RING, ring-finger domain; SRR, serine-rich region.

RNF43 was first reported as an oncoprotein that is highly expressed in CRCs (Yagyu *et al*. 2004). It strongly suppresses Wnt signals in healthy conditions and various genetic mutations in *RNF43* are identified in many cancers; thus, *RNF43* mutations are thought to be deeply involved in carcinogenesis as a tumor suppressor gene. Indeed, compound mutant mice lacking both RNF43 and ZNRF3 rapidly form tumorigenic expansion of ISCs (Koo *et al*. 2012). In particular, *RNF43* mutations are frequently identified in colorectal, pancreatic, and bile duct cancer, but strangely, there are not many genetic mutations in the homolog *ZNRF3* (Wu *et al*. 2011; Ong *et al*. 2012; Giannakis *et al*. 2014; Bond *et al*. 2016). RNF43 mutations have been identified in 11.4% of total CRCs (COSMIC database, https://cancer.sanger.ac.uk/cosmic/). The nature of CRCs is known to be significantly different whether tumors develop on the right or left side of the colon (Fig. 5, *left*) (Tsukiyama *et al*. 2021). The famous *APC* mutations are common in left-sided microsatellite stable (MSS) CRCs that arise in tissues developmentally originating from the hindgut, but this mutation is not frequently observed in right-sided microsatellite instability-high (MSI-Hi) CRCs. Conversely, right-sided MSI-Hi CRCs that arise in tissues derived from the midgut often have mutations in the *RNF43* gene instead of *APC* (Yaeger *et al*. 2018; Salem *et al*. 2020). Rather, *APC* and *RNF43* mutations are reported to be mutually exclusive, and they rarely coexist simultaneously (Giannakis *et al*. 2014; Yaeger *et al*. 2018; Fennell *et al*. 2020). These facts indicate that both mutations in RNF43 and APC activate Wnt signals in the same direction, and either one alone can provide sufficient conditions equally to initiate tumorigenesis. Mutations in RNF43 are diverse, including truncating mutations with missense and frameshift or substitution mutations with missense in both extracellular and intracellular regions. These numerous mutations are roughly classified into four groups depending on how mutations affect cells (Fig. 5, *right*).

### Latent mutations, which do not affect Wnt signaling

Most of the missense mutations in the intracellular domain and small deletions in the C-terminal side, such as R659fs mutation that is frequently observed in MSI-Hi, belong to this group (Fig. 5, *right*, *second from top*). These mutations have at least little or no impact on the function of RNF43 to ubiquitinate and degrade Fzds (Tu *et al*. 2019; Li *et al*. 2020; Cho *et al*. 2022). Latent mutants behave similarly to wild-type normal RNF43, so they can upregulate surface Fzd level and facilitate the Wnt signal in the presence of Wnt ligands in a Rspo-dependent manner (Fig. 3). However, whether these mutants are completely normal and fully retain the original function as a tumor suppressor remains unclear because RNF43 may be involved in other signaling pathways that affect tumorigenesis. Several molecules that interact with RNF43, including HAP95 and PSF/p54nrb, have been reported but with unclear biological significance of the binding (Miyamoto *et al*. 2008; Sugiura *et al*. 2008). In the future, the functions of RNF43, other than regulating Wnt signaling, should be dissected with these latent mutants.

### Dominant-negative (DN) mutations that facilitate Wnt signaling

Most missense mutations to the intracellular domain do not provide the phenotype in Wnt facilitation, except for phospho-switches or direct mutations to RING, which is essential for ubiquitin ligase activity, as described above (Tsukiyama *et al*. 2020; Yu *et al*. 2020). However, extracellular substitution mutations, such as I48T, S82S, and R127P, sometimes cause a DN effect on RNF43. RNF43 is matured via intracellular trafficking going through ER, Golgi, and MVB/SV to the cell surface (Fig. 4). These extracellular mutations are reported to disrupt RNF43 intracellular trafficking and cause its localization to stack in the ER (Tsukiyama *et al*. 2015). Kinases, including CK1, cannot phosphorylate mutant RNF43 trapped in the ER, so the phosphorylation switch is not turned on (Fig. 4, *right lower*). These not-phosphorylated and not-activated mutant proteins are expressed as full-length RNF43 proteins and are not only present as inactive proteins but also inhibit functional wild-type RNF43 and ZNRF3 that still express in cells because RNF43 forms homodimer and heterodimer with ZNRF3 (Zebisch et al. 2013; Tsukiyama *et al*. 2020; Toh *et al*. 2023). Remarkably, the R127P oncomutant was activated and functionally recovered to a tumor suppressor gene when a 3SD substitution was introduced into the phospho-switch of the R127P mutant (turning the switch to the ON) although it remained expressed in the ER (Tsukiyama *et al*. 2020). Therefore, the phospho-switch may be regulated in an intracellular localization-dependent manner. Cells that express mutant RNF43 completely lose the ability to degrade Fzds by both RNF43 and ZNRF3, causing surface Fzd accumulation and the hyper-sensitive state to Wnt ligands (Jiang *et al.* 2013). Therefore, those Wnt ligand–dependent hyper-activating cells are strikingly sensitive to PORCNi which eliminates Wnt proteins from extracellular space (Fig. 5, *right*, *third from top*) (Koo *et al*. 2015). Conversely, those cells do not respond to Rspo treatment, because excess Fzds in these cells do not depend on the substrate-switching by the LGRs-Rspo-RNF43 association but originated from the DN effect of mutant RNF43 itself.

### LOF mutations accelerate Wnt signaling

Recently, the second most frequent R117fs truncating mutation of RNF43 after G659fs was reported to facilitate Wnt signaling (Giannakis *et al*. 2014; Cho *et al*. 2022). This short RNF43 that lacks most of the essential motifs, including TM and RING, might interact with Fzds and prevent its ubiquitination and internalization, while retaining only partial PA domain (Fig. 5, *right*, *fourth from top*). Wnt facilitation with RNF43 (R117fs) mutants leads to Fzd accumulation and the hyper-sensitive state to Wnt ligands, thereby behaving similarly to DN mutants above, also showing PORCNi sensitivity and Rspo independency. However, whether the R117fs mutation acts as LOF or DN remains unclear. The effect of RNF43 (R117fs) that interacts with Fzds to block the binding of Fzds to endogenous wild-type and functional RNF43 and ZNRF3 may be DN rather than LOF. Further research is needed to investigate the molecular mechanism of how RNF43 (R117fs) accumulates Fzds and facilitates the Wnt signal.

### Gain of functional (GOF) mutations activate Wnt signaling

The recently reported R519X mutation causes aberrant Wnt signal activation in a Wnt ligand–independent manner, unlike the R659fs mutation, which does not show an impact on Wnt signals (Spit* et al*. 2020). This truncation mutation strongly stabilizes the binding of RNF43 to CK1 much more than that of wild-type type, resulting in constitutive membrane targeting of CK1 despite the absence of Wnt ligands (Fig. 5, *right*, *bottom*). Membrane localization of CK1 causes direct β-catenin accumulation through the destruction complex inactivation similar to the presence of Wnt ligands. The β-catenin accumulation and Wnt signal activation induced by R519X mutation do not accompany Fzd accumulation, so cells expressing this mutant do not show any Wnt dependency and sensitivity to PORCNi.

As described here, RNF43 is known to have a wide variety of mutations, and the changes in cellular properties resulting from these mutations also vary. In particular, all three types of mutations, namely DN, LOFs, and GOFs, facilitate or activate Wnt signaling and function as oncogenes (Fig. 5, *right*). Therefore, for CRCs with RNF43 mutations, the selection of drugs used should be considered depending on the type of RNF43 mutation (Tsukiyama *et al*. 2021). Here, we showed some of the representative mutations, but a study comprehensively describes for a huge number of mutations regarding Wnt signal facilitation/activation and the presence or absence of sensitivity to PORCNi; hence, please refer to this informative paper for the nature of RNF43 mutations (Yu *et al*. 2020).

## Versatile roles of RNF43 in multistep carcinogenesis

**Figure 6.**
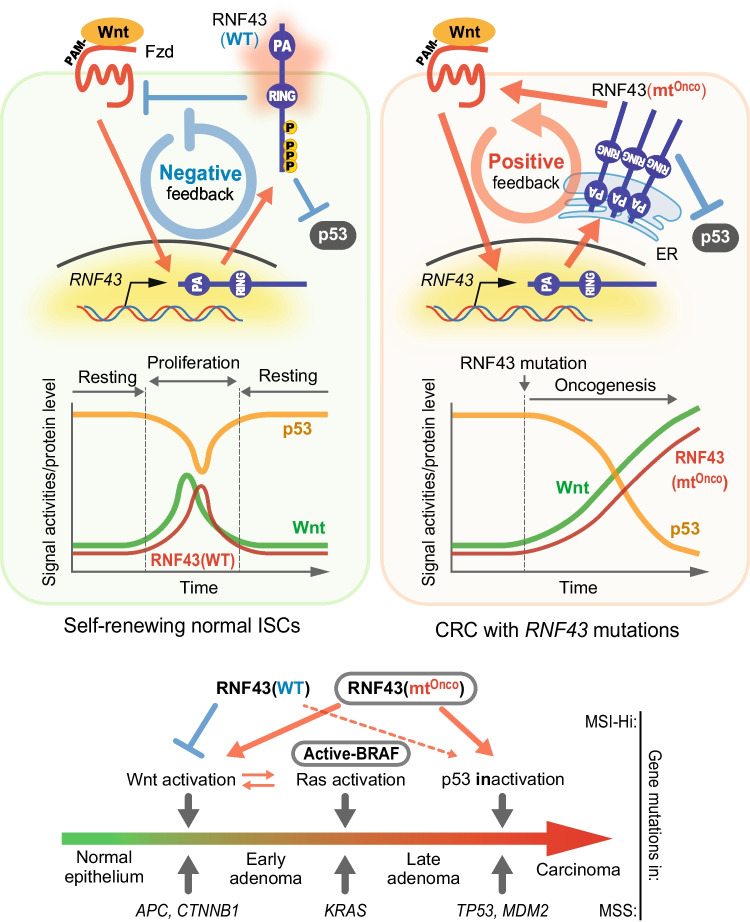
Diverse roles of RNF43 mutations in multistep carcinogenesis. Transient Wnt activation induces wild-type RNF43 in normal cells. RNF43 starts to suppress Wnt signaling via Fzd degradation, but it also suppresses the p53 pathway simultaneously (*upper left*, *diagram*). Attenuated Wnt signal reduces RNF43 expression to release p53 suppression, then returns to the resting state. Stem cells can self-renew only in the small window with hi-Wnt and low-p53 state (*upper left*, *graph*). Meanwhile, initial Wnt activation in CRCs even weakly induces oncogenic RNF43 and accelerates/amplifies Wnt signaling by forming positive feedback (*upper right*, *diagram*). Excess Wnt signal activity and p53 suppression may cause cells to proliferate out of biological control since these oncogenic RNF43 still suppress p53 (*upper right*, *graph*). Therefore, the oncogenic mutations of RNF43 in MSI-Hi CRC may simultaneously take the place of two mutations of APC and TP53 of MSS CRC (*bottom*). mt^Onco^, oncomutant.

Although RNF43 and ZNRF3 fine-tune Wnt signaling activity for maintaining homeostasis by regulating receptor expression, these ubiquitin ligases are also sensitive target genes of Wnt signaling (Takahashi N. *et al.* 2014; Tsukiyama *et al*. 2015). When Wnt signaling is activated, the β-catenin-Tcf/Lef transcriptional complex is formed in the WREs on the intron of the *RNF43* locus to initiate RNF43 expression. Sequentially, induced functional RNF43 protein terminates Wnt signaling as described, thereby forming a negative feedback circuit (Fig. 6, *upper left*). *RNF43* plays the role of a tumor suppressor as one of the gatekeepers that prevents excessive Wnt signaling. However, once *RNF43* acquires genetic mutations arising DN, LOF, or GOF that activate Wnt signaling (Fig. 5, *right*), active Wnt signal by those mutations induces the expression of further mutants, thereby reversing the feedback loop to positive (Fig. 6, *upper right*). Hence, the signal is amplified by positive feedback, leading to a runaway of Wnt signaling, rather than signal termination, despite the weak initial activation with a small number of Wnt ligands. To put it simply, a genetic mutation in *RNF43* causes the gatekeeper to switch sides and sell the Wnt signal to cancer.

Furthermore, RNF43 has been reported to suppress not only Wnt signaling but also downstream of a famous tumor suppressor gene known to be a guardian of the genome, p53 (Shinada *et al*. 2011; Nailwal *et al*. 2015; Xie *et al*. 2015; Tsukiyama *et al*. 2020). A clear mechanism has not yet been shown for how RNF43 suppresses the p53 pathway. These reports show inconsistent results, including that RNF43 directly degrades p53 by ubiquitination or it does not affect p53 stability but reduces its transcriptional activity (Shinada *et al*. 2011; Nailwal *et al*. 2015). However, all these studies, regardless of the molecular mechanism, at least revealed the same result that RNF43 suppresses Wnt signaling, although a recent report indicated the unlikely involvement of RNF43 in the p53 pathway (Li *et al*. 2020). RNF43 is expressed after a short delay when Wnt signal is activated in normal cells (Fig. 6, *upper left*). Induced RNF43 suppresses p53 as well as Wnt signaling simultaneously, so RNF43 expression is rapidly downregulated by Wnt inactivation. This sequence releases p53 repression by RNF43, then returning to the steady state as well as before Wnt activation. This steady state could be speculated as the dormant/quiescent phase of stem cells in the niche, and the window for stem cell self-renewal is allowed only during the period when RNF43 expression and p53 suppression occur simultaneously due to Wnt activation. Meanwhile, the expression of mutant RNF43, as well as Wnt signaling, upregulates at an accelerating rate once the positive feedback circuit activates in cancer cells carrying oncogenic RNF43 mutations (Fig. 6, *upper left*). The ability of RNF43 to suppress p53 between wild-type and oncogenic mutant demonstrated no significant difference; thus, p53 activity decreases as RNF43 expression increases in inverse proportion (Tsukiyama *et al*. 2021). Therefore, an initially weak Wnt activation later triggers both excess Wnt signaling activation and strong p53 repression simultaneously when RNF43 gets oncogenic mutations. These changes in cellular properties act positively for tumor initiation and progression.

Constitutive activation of Wnt signaling by gene mutations in *APC* or *CTNNB1* changes normal cells into early adenomas in the first step of carcinogenesis in the classic multistep carcinogenesis/tumorigenesis model of MSS CRC presented by Bert Vogelstein (Fearon and Vogelstein 1990). These mutations confer on normal and organized cells the ability to escape from biological regulation and proliferate cells autonomously. In the second step, positive feedback is established between the Wnt pathway and the Ras-MAPK pathway to accelerate and stabilize each other’s signals when the Ras pathway is constitutively activated by the mutations in *KRAS* or *BRAF* (Karni *et al*. 2005; Thornton *et al*. 2008; Ji *et al.* 2009; Jeong *et al*. 2012; Jeong *et al*. 2018). Adenoma then acquires to proliferate more aggressively, develops further, and progresses to the late adenoma stage. In the third step, p53 pathway inactivation due to mutations in *TP53* or *MDM2* transforms adenoma into carcinoma by immortalizing them. This mutation set frequently includes a pair of *RNF43* and *BRAF* mutations in MSI-Hi CRC, whereas the combination of *APC*, *KRAS*, and *TP53* mutations is common in MSS CRC as explained (Bond *et al*. 2016; Yan *et al.* 2017; Fennell *et al*. 2020; Salem *et al*. 2020). Mutations in *RNF43* are also mutually exclusive with those in *TP53* as has been previously reported to be similar to the case for *RNF43* and *APC* (Tsukiyama *et al*. 2020). These findings enabled us to hypothesize a hypothesis that the *RNF43* mutations compensate not only for *APC* in Wnt activation but also for the *TP53* mutation for cell immortality. Experimentally, only two mutations of *RNF43* and *KRAS* were sufficient for carcinogenesis (Tsukiyama *et al*. 2020). This study revealed that mutations in RNF43 simultaneously achieve the first and third steps of multistep carcinogenesis; thus, activation of just one more Ras pathway (second step) is sufficient for completing all steps of carcinogenesis.

These studies revealed a similar mechanism of carcinogenic progression between MSI-Hi and MSS CRCs, although the gene set is different between these distinguished CRC groups. Therefore, a complete understanding of the original RNF43 function and all the events caused by each genetic mutation including not only Wnt but also other signaling pathways will be important for developing the therapy of all cancer types carrying *RNF43* mutations (Elez *et al*. 2022; Quintanilha *et al*. 2023).

## Concluding remarks and future perspectives

The regulatory mechanism of Wnt receptors by RNF43 and the overall framework for its degradation are now roughly understood. However, the detailed functions of RNF43 in various biological reactions to maintain homeostasis and its functional change by genetic mutations remained unknown.

In particular, RNF43 was believed to function differently to suppress Wnt signaling at another point of the signaling cascade other than its membrane function. A report indicated that RNF43 suppresses Wnt signals by interacting with Tcf4 on the nuclear membrane and prohibiting its nuclear entry (Loregger *et al *. 2015). Indeed, when exogenous epitope-tagged RNF43 is detected by antibodies (Abs) for these tags, or endogenous RNF43 is stained with an anti-RNF43 Abs in cancer cells, some signals were observed around the nuclear membrane (Nailwal *et al*. 2015; Tsukiyama *et al*. 2015; Neumeyer *et al*. 2021). Biochemical fractionation also indicated that exogenous RNF43 is present in the nuclear fraction (Tsukiyama *et al*. 2021). However, endogenous RNF43 protein detection is now thought to be difficult due to their low expression (detectable by immunoblotting only after enrichment using immunoprecipitation form many cells) and/or rapid turnover (half-life within 90 min) (Tsukiyama *et al*. 2015, 2021). Even when HA or FLAG tags, which allow much more sensitive and specific detection than antibodies against endogenous proteins, are directly knocked into RNF43 locus by genome editing, at least, the evidence that RNF43 expresses in nuclear membrane was not obtained (Tsukiyama *et al*. 2021; Li *et al*. 2023). Additionally, a more recent report of RNF43 has revealed that the nuclear localization observed when stained with most homemade or commercially available Abs against RNF43 protein was likely to be nonspecific signals (Li *et al*. 2023). The inability to detect an original expression of endogenous RNF43 under the biological condition remains a disadvantage in understanding the exact function of RNF43 and remains an unsolved issue since RNF43 was identified.

Another question is where RNF43 ubiquitinates Fzds in the cell. RNF43, which is localized in the ER immediately after synthesis, and mutants stuck in the ER due to oncogenic mutations are not yet activated by the phospho-switch and thus do not ubiquitinate Fzd because they are not activated yet. Fzd and RNF43 take similar intracellular transporting pathways; thus, opportunities for their interaction and ubiquitination are expected at any point within the cell after the activation as previously indicated (Tsukiyama *et al*. 2021). However, whether RNF43 is activated and ubiquitinates Fzds at the TGN later than ER, at secretory granules, or at the cell surface remains unclear. Therefore, it is essential not only to understand the correlation between the localization and the phosphorylation of RNF43 but also to clarify its binding fashion with Fzd. The fact that RNF43/ZNRF3 can widely degrade various members of the Fzd family suggests that it is via conserved regions on each protein or a common mechanism. To date, two theories have been proposed: RNF43 can degrade many Fzds because (1) Dvl bridges the binding between RNF43 and Fzd; and (2) the conserved extracellular CRD of Fzds directly binds to the extracellular PA domain or TM region of RNF43 (Jiang *et al.* 2015; Tsukiyama *et al*. 2015; Spit *et al*. 2020). However, we have not found a clear answer to it yet. Recent reports provided inconsistent results to those proposed models revealing that a part of Dvl-induced degradation of Fzds does not require RNF43/ZNRF3 or that the PA domain of RNF43 is not essential for Fzd degradation (Zeng *et al*. 2018; Radaszkiewicz and Bryja 2020). Hopefully, further studies by many groups will clarify the exact molecular mechanism in which RNF43/ZNRF3 recognizes and degrades Fzds to make a consensus in the community.

Furthermore, Fzds are thought to be the only RNF43 substrate. RNF43 suppresses p53, which does not appear to be ubiquitinated; thus, it might not be defined as a substrate strictly. However, a protease-activated receptor 2 (PAR2) has been identified recently as a novel RNF43 substrate in CRC (Nag *et al*. 2023). Elucidating how RNF43 recognizes this new substrate can provide us a hint for understanding the binding of RNF43 to Fzds. However, the commonly conserved amino acid in Fzds and the sequences of PAR2 demonstrate no significant similarity; thus, Fzd or PAR2 possibly interacts with RNF43 via the large structure, such as the inside of the transmembrane region consisting of seven small TM motifs. Further identification of novel substrates can be expected by analogy with the many-to-many correspondence between ubiquitin ligases and substrates. Additionally, strategies for anti-cancer treatment utilizing RNF43/ZNRF3 ubiquitin ligases are being developed at present. Recently, several strategies for cancer therapy were developed for degrading disease-relevant target receptor proteins, including IGF1R, EGFR, and PD-1, using RNF43/ZNRF3 with antibodies (AbTACs/PROTACs, PROTAB) or nanobodies (REULR), and appear to be effective in their membrane clearance (Marei *et al*. 2022). Another report demonstrated that an artificial chimera protein with Rspo furin domain for ligase binding and extracellular domain of PD-1 can degrade the target protein PD-L1 and induce T cell activation to suppress tumor growth (ROTACs) (Sun *et al.* 2023). Therefore, further development of research in the field of Wnt/RNF43 is expected to guide to the application in clinical therapy of cancers in the future.

## Data Availability

Data sharing is not applicable to this article as no new data were created or analyzed in this reviewing article.
